# Circulating miRNAs as Auxiliary Diagnostic Biomarkers for Multiple Myeloma: A Systematic Review, Meta-Analysis, and Recommendations

**DOI:** 10.3389/fonc.2021.698197

**Published:** 2021-07-08

**Authors:** Yunhui Xiang, Liuyun Zhang, Pinpin Xiang, Juan Zhang

**Affiliations:** ^1^ School of Medicine, University of Electronic Science and Technology of China, Chengdu, China; ^2^ College of Medical Technology, Chengdu University of Traditional Chinese Medicine, Chengdu, China; ^3^ Department of Laboratory Medicine, Sichuan Provincial People’s Hospital, University of Electronic Science and Technology of China, Chengdu, China

**Keywords:** biomarker, diagnosis, meta-analysis, microRNAs, multiple myeloma

## Abstract

Multiple myeloma (MM) is a hematologic malignancy characterized by aberrant expansion of monoclonal plasma cells with high mortality and severe complications due to the lack of early diagnosis and timely treatment. Circulating miRNAs have shown potential in the diagnosis of MM with inconsistent results, which remains to be fully assessed. Here we updated a meta-analysis with relative studies and essays published in English before Jan 31, 2021. After steps of screening, 32 studies from 11 articles that included a total of 627 MM patients and 314 healthy controls were collected. All data were analyzed by REVMAN 5.3 and Stata MP 16, and the quality of included literatures was estimated by Diagnostic Accuracy Study 2 (QUADAS-2). The pooled area under the curve (AUC) shown in summary receiver operating characteristic (SROC) analyses of circulating miRNAs was 0.87 (95%CI, 0.81–0.89), and the sensitivity, specificity, positive likelihood ratio (PLR), negative likelihood ratio (NLR), and diagnostic odds ratio (DOR) were 0.79, 0.86, 5, 0.27, 22, respectively. Meta-regression and subgroup analysis exhibited that “miRNA cluster”, patient “detailed stage or Ig isotype” accounted for a considerable proportion of heterogeneity, revealing the importance of study design and patient inclusion in diagnostic trials; thus standardized recommendations were proposed for further studies. In addition, the performance of the circulating miRNAs included in MM prognosis and treatment response prediction was summarized, indicating that they could serve as valuable biomarkers, which would expand their clinical application greatly.

**Systematic Review Registration:**

https://www.crd.york.ac.uk/prospero/display_record.php?RecordID=234297, PROSPERO, identifier (CRD42021234297).

## Introduction

Multiple myeloma (MM), the second most common hematological malignancy (1), develops from monoclonal gammopathy of undetermined significance (MGUS) and smoldering multiple myeloma (SMM), through the malignant transformation of long-lived plasma cells deriving from memory B cells and plasma-blasts (2). Normal interactions between plasma cells and their environment (the bone remodeling chambers) enable the stability of normal plasma cell genotypes and phenotypes, which may be disrupted by multiple factors at a precursor stage of MM (MGUS, pre-MGUS state) leading to MM tumorigenesis (3). The risk of progression for MGUS is about 1% yearly and 10% for SMM in the first 5 years (4). Early treatment is of benefit, inhibiting disease progression but with non-negligible side effects (5), and a novel biomarker with high accuracy for early diagnosis is imperative.

As a member of non-coding RNAs, miRNAs are small RNA molecules that function as negative regulators of gene expression. The decrease or increase of specific miRNAs in MM is associated with the dysregulation of the target gene expression, reflecting their impact on tumor-suppression or promotion (6, 7). Circulating miRNAs have been explored as valuable tools for various tumor diagnoses and prognoses by several studies (8). Similarly, the diversification of circulating miRNA expression in MM has been investigated, indicating that miRNAs could serve as potential diagnostic biomarkers (9, 10). However, not every miRNA was eligible, and there was significant heterogeneity among the findings. We updated a meta-analysis to address whether the circulating miRNAs could be promising biomarkers for early detection of MM with the latest evidence and try to resolve the problems that contributed to heterogeneity in previous studies.

## Materials and Methods

This systematic review and meta-analysis was conducted following the guidance of the PRISMA 2020 Statement: an Updated Guideline for Reporting Systematic Reviews (11), and registered on PROSPERO prior to the start.

### Literature Search

Multiple databases (Cochrane Library, PubMed, Embase, Web of Science, SinoMed, CQVIP database, Wan Fang database, China National Knowledge Infrastructure (CNKI), and Clinical Trials.gov) were systematically searched for related studies and essays published in English up to Jan 31, 2021. Subject headings and all the free words of ‘multiple myeloma’, ‘microRNAs’, ‘sensitivity’, ‘specificity’, ‘predictive value’, ‘accuracy’, ‘diagnostic’, and ‘AUC’ were applied for the regular advanced search. To minimize search omissions, we searched ScienceDirect and ResearchGate and pored over the reference list of the articles cited in this review to look for potential studies.

### Study Selection

Studies that met the following criteria were included: 1) all the MM patients were diagnosed according to standard diagnostic criterion; 2) control subjects were analyzed concurrently; 3) miRNA measured by qRT-PCR and the process clearly described; 4) same outcome: sensitivity, specificity, AUC; 5) specimens are limited to serum or plasma; and 6) sample size was given. The exclusion criteria were as follows: 1) different control groups; 2) reviews and meta-analysis; 3) abstracts, editorials, conference papers, and letters without valid data; 4) repeated articles; 5) not conducted on humans; 6) sample size was insufficient; 7) non-English literature. To avoid any selection bias, two independent authors decided whether to include or exclude a study when they reach a consensus; otherwise, a third author reviewed the article again and resolved the disagreement.

### Quality Assessment of Literature and Data Extraction

The quality of the selected studies was assessed according to QUADAS-2 (Quality Assessment of Diagnostic Accuracy Studies-2) on RevMan (version 5.3, London, UK, RRID: SCR_003581) by two authors and illustrated in a graph. Also, basic information from eligible articles/supplementary materials (first authors, publication year, specimen, sample size, miRNAs, expression mode, sensitivity, specificity, AUC, patient information including Ig isotype, stage, study design, gender structure, and age range) was extracted by two authors independently using a standardized form and then reviewed by a third author in detail.

### Statistical Analysis

The data of true positives (TPs), false positives (FPs), true negatives (TNs), and false negatives (FNs) of 32 miRNAs extracted from 11 individual studies were analyzed in REVMAN 5.3 and STATA (version MP16, Texas, USA, RRID : SCR_012763). The sensitivity, specificity, positive likelihood ratio (PLR), negative likelihood ratio (NLR), diagnostic odds ratio (DOR), and area under the curve (AUC) were pooled with the random effects meta-analysis model. Total diagnostic accuracy was assessed by summary receiver operating characteristic (SROC) curve with the sensitivity and specificity data of each qualified study. To evaluate the existence of heterogeneity, I^2^ over 50% and/or P-value of Q-test under 0.05 were set as statistically significant. Meta-regression was executed with univariable model and multivariate model respectively. Subgroup analyses were employed to dissociate the heterogeneity among the studies using the random-effects inverse-variance model with DerSimonian–Laird estimate of tau². All *P*-values less than 0.05 were considered statistically significant.

## Results

### Literature Selection, Quality Assessment, and Study Characteristics

**Figure 1:**
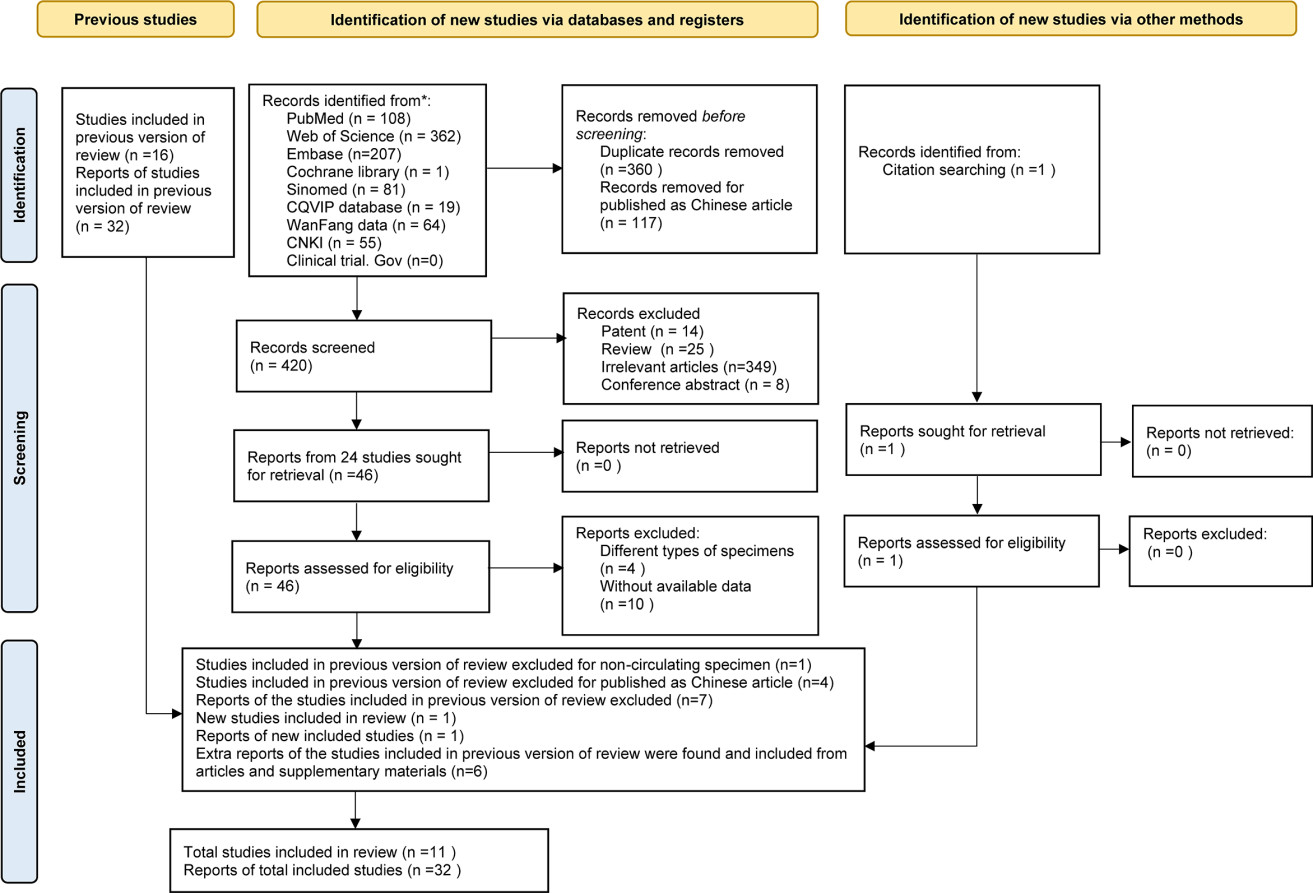
Study selection flow diagram.

As the study search and selection process show in **Figure 1**, a total of 898 articles were initially obtained; 897 studies were identified from Cochrane Library, PubMed, Embase, Web of Science, SinoMed, CQVIP database, Wan Fang Data, China National Knowledge Infrastructure (CNKI), and Clinical Trials.gov, and 1 was acquired from reference lists of articles cited in this paper. All of the articles were imported into Zotero. After carefully screening for the title. abstract, and full-text, 117 Chinese articles, 360 duplicates, 14 patent, 25 reviews, 8 conference abstracts, 349 with irrelevant themes, 5 with different types of specimen, and 10 without available data were excluded. Finally, 32 miRNAs from 11 articles were included for this meta-analysis.

In comparison to the previous version of review (10), one report of a study for non-circulating specimen (12) and seven reports of four articles published in Chinese were excluded; and one report of a new study was included (13). The main information of circulating miRNA reports extracted from the qualified studies is displayed in **Table 1**. Among these, six extra reports of the studies included in the previous version of review were found from the articles and supplementary materials (14, 18, 21, 22). Characteristics of MM patients and healthy controls are available in **Table 2**. All the eligible articles were published before Jan 31, 2021 containing 627 MM patients and 314 healthy controls. The expression levels of miRNAs in serum (n = 27) or plasma (n = 5) were detected by qRT-PCR. Of these 32 studies, three studies evaluated miRNA clusters, whereas the others evaluated individual miRNA.

**Table 1:** Characteristics of studies and the diagnostic power of miRNAs.

No.	Author	Country	Year	Sample size		Sample	miRNA	Expression	Diagnostic power		
				MM	HC				AUC	SENC	SPEC
1	Li J (14)	China	2020	23	18	serum	miR-134-5P	↑	0.812	0.870	0.667
2						serum	miR-107	↑	0.766	0.564	0.889
3						serum	miR-15a-5p	↑	0.804	0.870	0.610
4	Gupta N (15)	India	2019	30	30	serum	miR203	↓	0.930	0.833	0.833
5						serum	miR143	↓	0.864	0.767	0.767
6						serum	miR144	↓	0.784	0.733	0.733
7						serum	miR199	↓	0.900	0.800	0.800
8	Shen X (16)	China	2017	71	46	serum	miR-4449	↑	0.885	0.789	0.913
9	JIANG Y (17)	China	2018	35	20	plasma	miR-125b-5p	↑	0.954	0.860	0.960
10						plasma	miR-490-3p	↑	0.866	0.600	0.850
11	Qu X (18)	China	2014	40	20	plasma	miR-483-5p	↑	0.745	0.580	0.900
12						plasma	miR-20a	↓	0.740	0.630	0.850
13	Kubiczkova L (19)	Czech	2013	103	30	serum	miR-744	↓	0.715	0.728	0.667
14						serum	miR-130a	↓	0.722	0.575	0.900
15						serum	miR-34a	↑	0.790	0.777	0.700
16						serum	let-7d	↓	0.804	0.641	0.867
17						serum	let-7e	↓	0.829	0.888	0.633
18	Yoshizawa S (20)	Japan	2011	62	21	plasma	miR-92a	↓	0.981	0.919	0.991
19	Jones Cl (21)	UK	2012	24	13	serum	miR-720	↑	0.911	0.872	0.923
20						serum	miR-1308	↓	0.892	0.821	0.923
21	Hao M (22)	China	2015	108	56	Serum	miR-4254	↑	0.926	0.793	0.985
22						Serum	miR-19a	↓	0.910	0.773	0.897
23						Serum	miR-92a	↓	0.830	0.724	0.869
24						Serum	miR-135b-5p	↑	0.810	0.667	0.833
25						Serum	miR-214-3p	↑	0.720	0.625	0.833
26						Serum	miR-3658	↑	0.720	0.714	0.667
27						Serum	miR-33b	↑	0.630	0.633	0.815
28	Y u J (13)	China	2014	40	30	serum	miR-202	↓	0.711	0.800	0.600
29	Sevcikova S (23)	Czech	2013	91	30	Serum	miR-29a	↑	0.832	0.880	0.700
**miRNA cluster**											
30	Kubiczkova L (19)	Czech	2013	103	30	serum	miR-34a+let7e	N/A	0.898	0.806	0.867
31	Jones Cl (21)	UK	2014	24	13	serum	miR-1308/miR-720	N/A	0.986	0.974	0.923
32	Hao M (22)	China	2015	108	56	Serum	miR-4254/miR-19a	N/A	0.950	0.917	0.905

AUC, area under the curve; HC, health control; MM, multiple myeloma; N/A, Not applicable; SENS, sensitivity; SPEC, specificity; ↑, increase; ↓, decrease.

**Table 2:** Characteristics of patients and health controls included.

Author	MM Ig Isotype	MM stage	Newly diagnosed or untreated	Cohort study	Gender (male, female)		Age (median, range)	
					MM	HC	MM	HC
Li J (14)	IgG: 9, IgA: 7, Light chain only: 7	D-S, I: 3; II 5; III: 15	NA	NA	16, 7	11, 7	66.5 (42–86)	65.6 (53–79)
Gupta N (15)	NA	ISS, I: 4; II 14; III: 12	YES	NA	17,13	22, 8	59 (33-76)	44 (33-55)
Shen X (16)	NA	NA	YES	YES	13, 10	NA	62 (39-86)	63 (40-76)
JIANG Y (17)	NA	D-S, I: 10; II 16; III: 19	YES	NA	23, 12	8, 12	59 (35-75)	(17-63)
Qu X (18)	IgG: 18, IgA: 10, IgD: 1, Light chain only: 10, non-secretory: 1	ISS, I: 7; II: 13; III: 20	YES	NA	23, 17	10, 10	59 (23-80)	60 (35-75)
Kubiczkova L (19)	IgG: 54, IgA: 28, IgD: 3, IgM: 2,Light chain only: 11	ISS, I: 35; II: 29; III: 39	YES	YES	51, 52	14, 16	66 (47-83)	55 (45-64)
Yoshizawa S (20)	IgG: 26, IgA: 12, IgD:3, B-J protein: 14, non-secretory: 4; plasma cell leukemia: 3	NA	YES	NA	NA	NA	NA	NA
Jones Cl (21)	NA	NA	NA	NA	12, 12	5, 8	73.5 (58-89)	47.7 (42-58)
Hao M (22)	IgG: 51, IgA: 29, IgD: 8, Light chain only: 18, non-secretory: 2	ISS, I: 18; II: 39; III: 50, not classified: 1.	YES	YES	NA	NA	(33-83)	52 (40-78)
Y u J (13)	IgG: 22, IgA: 13, IgD: 5	NA	NA	NA	25, 15	18, 12	62 (35-87)	63 (40-86)
Sevcikova S (23)	IgG: 46, IgA: 22, IgD: 2 Light chain only: 16, non-secretory: 2, Biclonal:1	ISS, I: 28; II: 32; III: 26, not classified: 5.	YES	YES	49, 42	14, 16	63.9 (41-48)	55.5 (45-64)

D-S, Durie-salmon stage; HC, health control; ISS: International Staging System stage; MM, multiple myeloma; NA, not available.

**Figure 2:**
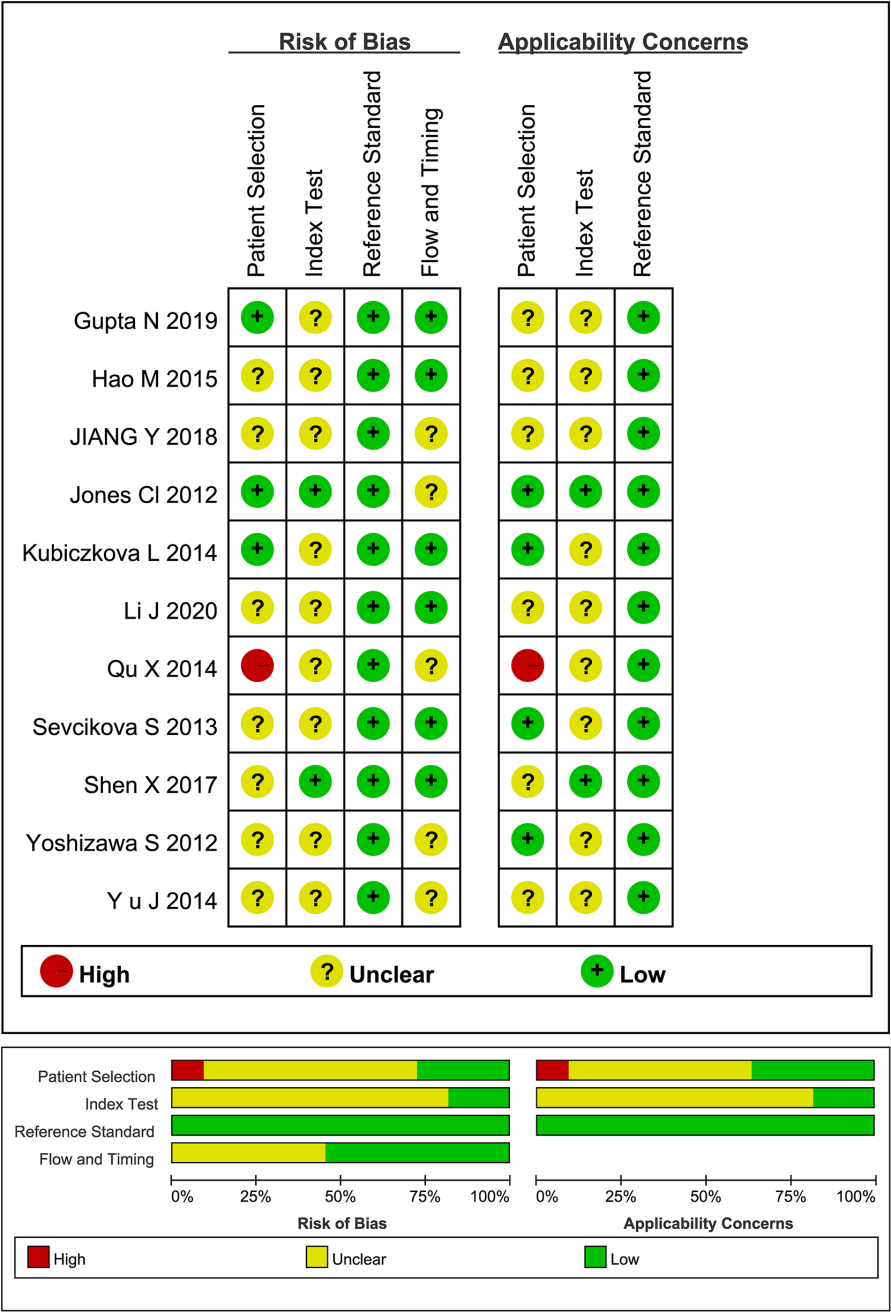
Quality assessment of qualified studies by QUADAS-2 tool.

Quality assessment of qualified literatures by QUADAS-2 tool reveal that the overall quality of the studies included was acceptable, but with a few unignorable flaws and uncertainties (**Figure 2**). Some of the literatures did not clarify the study design, the patient information (stage/Ig isotype/prior to any treatment), and whether there was an appropriate time interval between the reference standard and miRNA detection. Besides, the absence of suspected cases in some of the literatures may lead to bias in the evaluation of diagnostic power.

### Diagnostic Performance Evaluation

**Figure 3:**
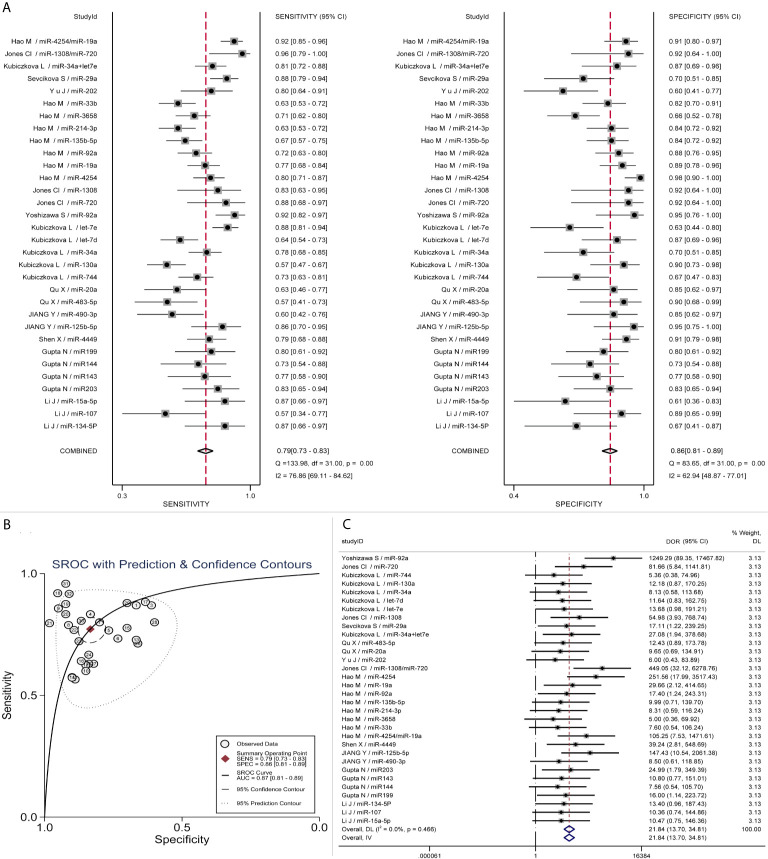
Pooled diagnostic parameters of all microRNA studies. **(A)** Forest plot of Sensitivity and Specificity; **(B)** SROC curve; **(C)** Forest plot of DOR.

All the data were included to calculate the combined diagnostic accuracy of miRNAs by STATA MP 16 software. As Forest plots present, the sensitivity and specificity of miRNAs in various studies were significantly heterogeneous (sensitivity: I^2^ = 76.86%, p < 0.01; specificity: I^2^ = 62.94%, p < 0.01). By recombining the data with random effect models, the pooled sensitivity and specificity were 0.79 (95%CI, 0.73–0.83) and 0.86 (95%CI, 0.81–0.89) respectively (**Figure 3A**). Spearman correlation analysis was performed on the Logit conversion values of sensitivity and false-positive rate to identify the source of heterogeneity, and no threshold effect was suggested (coefficient = −0.0817, p = 0.657). Based on the SROC curve, the pooled AUC of miRNAs in MM diagnosis was 0.87 (95%CI, 0.81–0.89), PLR 5 (95%CI, 4–6), NLR 0.27 (95%CI, 0.23–0.33) (**Figure 3B**). The pooled DOR was 22 (95%CI, 14–35) using a random effect model (**Figure 3C**).

**Figure 4:**
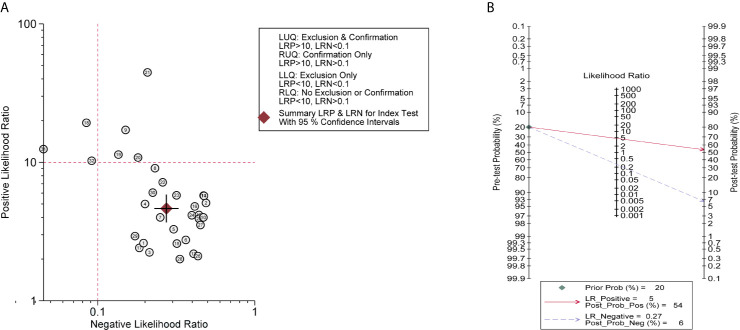
Clinical utility of circulating miRNAs. **(A)** Summary LRP & LRN for Index Test showed that a few miRNAs (No. 9/18/19/20/21/31/32) had relatively good clinical diagnostic value. LLQ, left lower quadrant; LRN, likelihood ratio negative; LRP, likelihood ratio positive; LUQ, left upper quadrant; RLQ, right lower quadrant; RUQ, right upper quadrant; **(B)** Fagan nomogram of Pre-test probability and post-test probability.

Summary LRP and LRN for Index Test are depicted in **Figure 4A**, demonstrating that a small number of miRNAs (No. 9/18/19/20/21/31/32) contribute to the confirmation or exclusion of MM, whereas the others appear to be under-achieving. The pre-test and post-test probabilities were evaluated by Fagan’s nomogram, presenting that when the prior probability was 20%, post-test probabilities were 54 and 6% for positive and negative circulating miRNAs in MM patients, respectively (**Figure 4B**).

### Sensitivity Analysis and Heterogeneity Exploration

**Figure 5:**
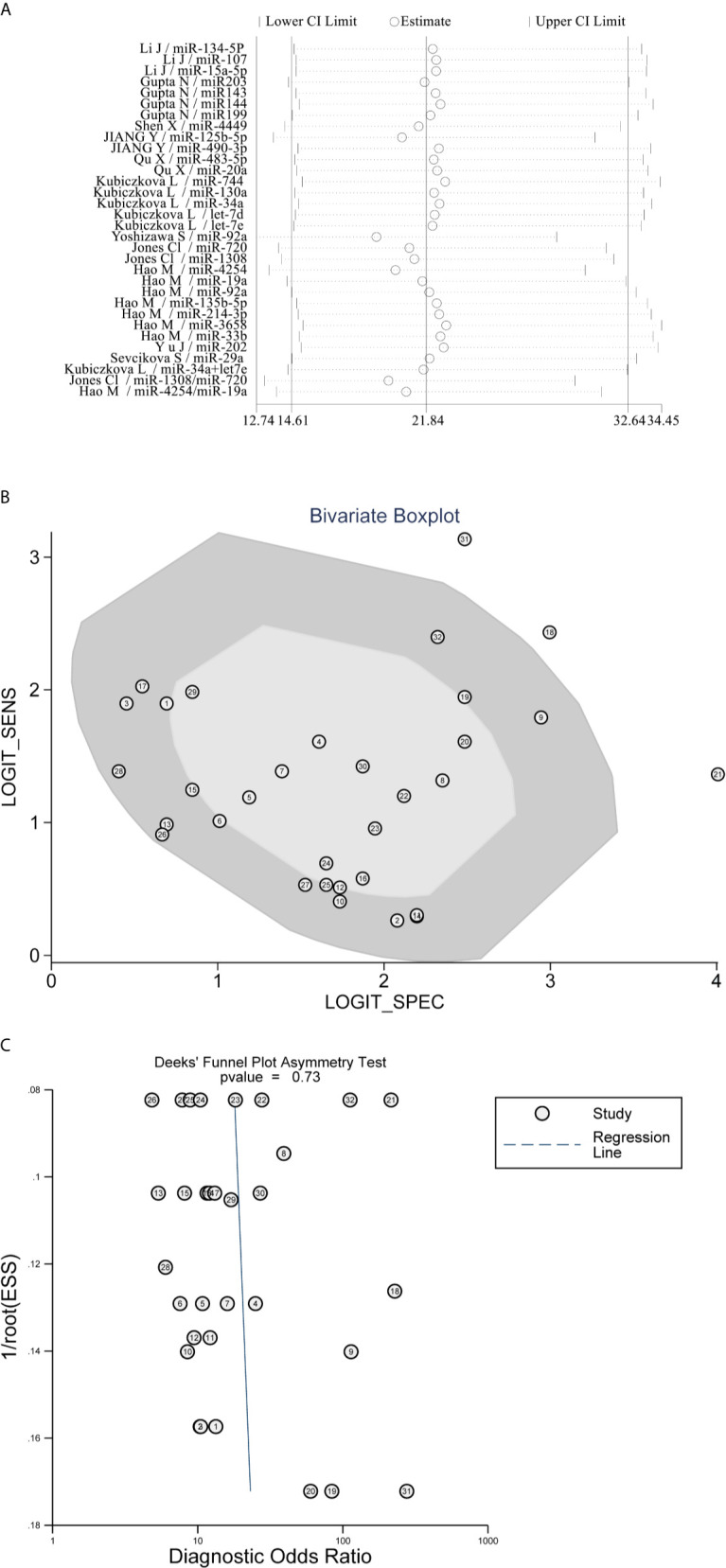
Sensitivity analysis and Heterogeneity exploration. **(A)** Sensitivity Analysis showed that the combination results were stable; **(B)** Bivariate Boxplot revealed that No.18/21/31 studies presented strong heterogeneity; **(C)** Deek’s Funnel Plot Asymmetry Test found no publication bias.

Then, a sensitivity analysis was performed to evaluate the stability of the combined DOR. After subtracting the No. 18 study with the maximal deviation, the DOR value changed from 22 to 19, presenting that there was no significant change in the results (**Figure 5A**). Similarly, other studies, excluding one, showed at the time consistent combinatorial DOR without significant fluctuations. However, the fixed-effect model had a greater influence on the pooled DOR than the random-effect model (DOR, 17 *vs*. 22), indicating that an improper data analysis method may have a great impact on the pooled results. Among these miRNAs, miR-4254 was the most promising diagnostic biomarker with 0.80 sensitivity (95%CI, 0.71–0.87), 0.98 specificity (95%CI, 0.90–1.0) and 252 DOR (95%CI, 18–3517). Bivariate boxplot revealed that some of the studies with relatively higher AUC (No.18/21/31) showed stronger heterogeneity (**Figure 5B**). Meanwhile, publication bias was not found by Deek’s funnel plot asymmetry test (P = 0.73) (**Figure 5C**).

**Figure 6:**
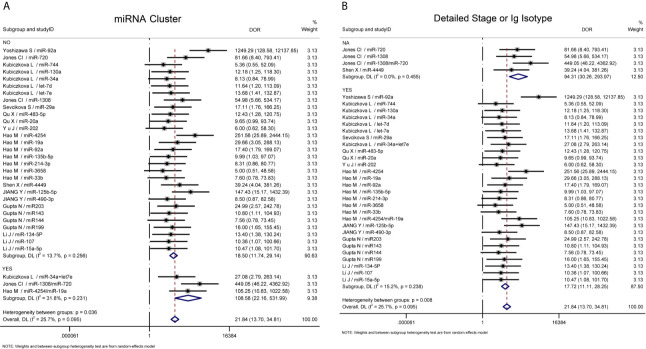
Subgroup analysis. **(A)** Subgroup analysis based on DOR sorted by “miRNA cluster”; **(B)** Subgroup analysis based on DOR sorted by “detailed stage or Ig isotype”. NA, not available.

Further heterogeneity analysis by meta-regression according to “miRNA cluster”, “ detailed stage or Ig isotype”, “newly diagnosed or untreated”, “determined cohort study design”, “sample size>30”, “serum or plasma” and “ethnicity” were conducted with univariable model, demonstrating that “miRNA cluster”, “detailed stage or Ig isotype” and “determined cohort study design” reached statistical significance. Subsequently, these three variables were analyzed by meta-regression with multivariate model, and there were significant differences between the “miRNA cluster” subgroups and the “detailed stage or Ig isotype” subgroups (**Table 3**). Then subgroup analyses based on sensitivity, specificity, AUC, and DOR were performed using the random-effects inverse-variance model with DerSimonian–Laird estimate of tau² to investigate the specific heterogeneity existing within and between subgroups, and the results are displayed in **Table 4**. The diagnostic parameters of miRNA clusters were better than the single miRNA subgroup, whereas the parameters of the detailed patient information subgroup and determined cohort study design subgroup were inferior than their contrasts. The DOR-based subgroup analysis results are shown in **Figure 6**.

**Table 3:** Meta-regression with univariable model and multivariate model.

Variable	Coef.	Std. Err	t	P>|t|	95% CI	Tau²	I-squared res	Adj R-squared	exp(b)
**Univariable model**									
miRNA cluster	1.77	0.76	2.32	0.028	[0.21, 3.33]	0.24	15.24%	48.02%	5.87
_cons	2.92	0.23	12.47	0.000	[2.44, 3.40]				18.5
Detailed stage or Ig isotype	-1.67	0.66	-2.52	0.017	[-3.02, -0.31]	0.20	12.92%	57.11%	0.19
_cons	4.54	0.62	7.31	0.000	[3.27, 5.81]				94.31
Cohort study	-0.46	0.48	-0.97	0.340	[-1.43, 0.51]	0.47	25.84%	-0.73%	0.63
_cons	3.31	0.34	9.84	0.000	[2.62, 4.00]				27.51
**Multivariate Model**						0.00	0.00%	100.00%	
miRNA profile	1.20	0.74	1.63	0.115	[-0.35, 2.65]				3.32
Detailed stage or Ig isotype	-2.42	0.74	-3.30	0.003	[-3.75, -0.63]				0.09
Cohort study	-0.39	0.42	-0.92	0.363	[-1.72, 0.22]				0.68
_cons	5.36	0.72	7.45	0.000	[4.01, 6.99]				213.70

Adj R-squared, Adjusted R-Squared; CI, confidence interval; Coef, coefficient; I-squared res, I-squared residual; Std. Err, standard error.

**Table 4:** Subgroup analysis.

Subgroup	SENS [95% CI]	SPEC [95% CI]	AUC [95% CI]	DOR [95% CI]
**miRNA profile**				
Single miRNA	0.77 [0.70, 0.82]	0.85 [0.80, 0.89]	0.84 [0.79, 0.88]	19 [12, 29]
I²/p-value	0.0%/0.99	6.8%/0.361	0.0%/0.928	13.7%/0.256
miRNA cluster	0.92 [0.77, 0.98]	0.90 [0.75, 0.97]	0.96 [0.87, 0.99]	109 [22, 532]
I²/p-value	38.1%/0.199	0.0%/0.909	21.1%/0.282	31.8%/0.231
P (between subgroup)	0.047	0.440	0.021	0.036
**Stage or Ig isotype**				
not detailed	0.89 [0.74, 0.96]	0.92 [0.81, 0.97]	0.93 [0.84, 0.98]	94 [30, 294]
I²/p-value	31.7%/0.222	0.0%/1.000	21.6%/0.281	0.0%/0.455
detailed	0.77 [0.70, 0.82]	0.84 [0.79, 0.89]	0.84 [0.79, 0.88]	18 [11, 28]
I²/p-value	0.0%/0.968	6.1%/0.373	0.0%/0.863	15.2%/0.238
P (between subgroup)	0.099	0.152	0.071	0.008
**Cohort study design**				
not detailed	0.81 [0.74, 0.87]	0.87[0.79, 0.92]	0.88 [0.82, 0.93]	28[13,60]
I²/p-value	34.4%/0.442	17.6%/0.252	14.1%/0.292	46.2%/0.022
detailed	0.7 [0.68, 0.83]	0.84 [0.77, 0.90]	0.83 [0.75, 0.88]	17 [10, 31]
I²/p-value	0.0%/0.951	0.0%/0.648	0.0%/0.936	0.0%/0.635
P (between subgroup)	0.340	0.649	0.185	0.346
**Overall**	0.79 [0.73, 0.83]	0.86 [0.81, 0.89]	0.87 [0.81, 0.89]	22 [14, 35]
I²/p-value	0.0%/0.839	0.0%/0.475	0.0%/0.670	25.7%/0.095

AUC, area under the curve; DOR, diagnostic odds ratio; SENS, sensitivity; SPEC, specificity.

### Circulating miRNAs in MM, MGUS, and SMM

In order to investigate the ability of the included circulating miRNAs to discriminate MM from MGUS/SMM, we summarized their distributional and differential diagnostic data among those disease states. Between the MM and MGUS groups, the levels of miR-20, miR-15a, and miR-92a were significantly different. In ROC analysis, the individual miRNAs did not exhibit substantial discriminative performance, which would be improved by the combination of miRNAs and other clinical parameters. The combination of miR-107, miR-15a-5p, and hemoglobin gained the best differential performance with AUC = 0.954 (14), and the combination of miR-1246 and miR-1308 ranked second with AUC = 0.725 (21). For MM and SMM, the expression level of miR-92a was significantly different, but the differential diagnostic value remains to be verified (20). For MGUS and SMM, Manier et al. found significant differences in the expression levels of miR-107, miR-92a, and miR-125a in circulating exosomes (24); however, the included serum or plasma miRNAs have not shown any differential diagnostic value (**Table 5**).

**Table 5:** Circulating miRNAs in MM, MGUS, and SMM.

Author	Sample size		miRNAs	Distributional or differential diagnostic data
**MM *vs* MGUS**	**MM**	**MGUS**		
Li J (14)	23	16	miR-134-5P	ROC: AUC=0.489 (p=0.909)
			miR-107	ROC: AUC=0.427 (p=0.441)
			miR-15a-5p	ROC: AUC=0.557 (p=0.549)
			miR-134-5P + miR-107 + miR-15a-5p	ROC: AUC=0.550 (p=0.095)
			mir-107 + mir-15a-5p + Hb	ROC: AUC=0.954 (p=0.000), sensitivity=0.913, specificity=0.917
Kubiczkova L (19)	103	57	miR-744	MM: 473, MGUS: 371, fold change between MM/MGUS: 1.27
			miR-130a	MM: 5618, MGUS: 6232, fold change between MM/MGUS: 0.90
			miR-34a	MM: 176, MGUS: 192, fold change between MM/MGUS: 0.92
			let-7d	MM: 1944, MGUS: 1863, fold change between MM/MGUS: 1.04
			let-7e	MM: 4222, MGUS: 3521, fold change between MM/MGUS: 1.20
Yoshizawa S (20)	62	22	miR-92a	One-way analysis of variance: p=0.0005
Jones Cl (21)	24	15	miR-720	ROC: AUC=0.528 (p=0.773)
			miR-1308	ROC: AUC=0.572 (p=0.453)
			miR-1246	ROC: AUC=0.628 (p=0.184)
			miR-1308/miR-720	ROC: AUC=0.597 (p=0.312)
			miR-1246/miR-1308	ROC: AUC=0.725(P< 0.05), sensitivity=0.792, specificity=0.667
**MM *vs* SMM**	**MM**	**SMM**		
Yoshizawa S (20)	62	8	miR-92a	One-way analysis of variance: p=0.0496
Zhang Z (25)	20	20	let-7d-5p	Mann‐Whitney U test and one‐way analysis of variance: p=0.354
			miR-20a-5p	Mann‐Whitney U test and one‐way analysis of variance: p=0.402
**MGUS *vs* SMM**	**MGUS**	**SMM**		
Yoshizawa S (20)	22	8	miR-92a	One-way analysis of variance: p=0.2959
Manier S (24)	4	4	miR-107	p < 0.05
			miR-92a	p < 0.05
			miR-125a	p < 0.05

AUC, area under the curve; Hb, hemoglobin; MGUS, monoclonal gammopathy of undetermined significance; MM, multiple myeloma; ROC, receiver operator characteristic curve; SMM, smoldering multiple myeloma.

## Discussion

Although survival of MM patients have been improved with the rapid advances of therapeutic strategy and supportive care, myriad patients still suffer from relapsed/refractory MM, entailing a reliable biomarker for early diagnosis. The remarkable impact of miRNA on protein expression is emerging with the discovery that about one-third of human encoding genes are regulated by miRNAs (26). Pioneering studies have found a mass of specific miRNAs carried by circulating microparticles significantly distinct from their maternal cells, depicting an interesting transfer pathway for the gene-regulating function of miRNAs from microparticles releasing cells to the target cells (27).

MicroRNAs transfer between MM plasma cells and bone marrow microenvironment, enabling the development and metastasis of malignancy through messenger RNA destruction or translation inhibition (28, 29). Circulating microRNAs have been applied in hematological diseases as diagnostic biomarkers due to their reliable stability and non-invasive properties (30). In recent years, the important role of circulating miRNAs in MM diagnosis has received considerable attention; however, the results vary and leave questions open (10). We updated this meta-analysis to figure out whether the circulating miRNAs could be promising means for early detection of MM with the latest evidence.

The diagnostic performance of miRNAs differed in this meta-analysis; among the top three individual miRNAs, miR-4254 was the highest [DOR = 252, 95% CI = (18,3517)] followed by miR-125b-5p [DOR = 147, 95% CI = (11,2061)], then miR-720 [DOR = 82, 95% CI= (6,1142)], whereas miR-3658 was the lowest [DOR = 5, 95% CI = (0.36,70)]. The miRNA cluster exhibited better diagnostic performance in subgroup analysis, with the combined parameters (sensitivity, 0.92; specificity, 0.90; AUC, 0.96; DOR 109) being far beyond individual miRNAs (sensitivity, 0.77; specificity, 0.85; AUC, 0.84; DOR 19).

The pooled sensitivity, specificity, AUC, PLR, NLR, and DOR were 0.79, 0.86, 0.87, 5, 0.27, and 22, respectively, consistent with the previous meta-analysis (10). Summary LRP and LRN for Index Test suggest a small number of studies have relatively high value for diagnosis conformation and/or exclusion. The Fagan nomogram of post-test probability also indicated that circulating miRNAs were of relatively good diagnostic value but still had room for improvement. Researchers found that the tests based on serum or plasma did not show significant differences, nor between ethnic groups; and our meta-analysis confirmed it.

However, the quality assessment of literatures presents a few ignorable flaws and uncertainties, which are somewhat inconsistent with the results of the previous meta-analysis (10), possibly because of the different versions of evaluation tools or the strictness in our assessment. We found that some of the literatures did not specify the study design, patient information (stage/Ig isotype/treatment information), or the time interval between the reference standard and miRNA detection. Besides, the absence of suspected cases in some of the studies may lead to bias in evaluation.

The combined diagnostic power did not fluctuate significantly in sensitivity analysis. The DOR value only changed from 22 to 19 even when the study with the greatest deviation was eliminated. To dissociate the sources of heterogeneity, a publication bias assessment was performed and no publication bias was found (p = 0.73). Inevitably, the exclusion of non-English literatures may also lead to selection bias. However, significant differences were found in the “miRNA cluster” and “detailed stage or Ig isotype” subgroups through meta-regression with univariable model and multivariate model (p < 0.05), but not in the “cohort study” and “newly diagnosed and untreated” subgroups, which may also be influenced by the limited number of studies included in each subgroup. Besides, no significant differences were found in the “specimen” and “ethnicity” subgroups, consistent with previous studies. These results reveal that the study design and the enrolment of patients and healthy controls may have an impact on the diagnostic value of the index to be assessed, and a standardized recommendation is imperative.

At present, the efficacy of early treatment determined by the free light chain ratio for the precursor-stage of MM (MGUS or SMM) to improve longevity and health-related quality of life is still unclear (31, 32), and distinguishing symptomatic multiple myeloma from those conditions is of great importance. In our summary, not much distributional or differential diagnostic evidence of circulating miRNAs in MM, MGUS and SMM were found. Although miRNA expression levels differed significantly, no individual miRNA exhibited excellent differential diagnostic ability, indicating that this area needs to be further explored with more meticulous design for subject enrolment. Moreover, a variety of diseases such as inflammation (33), cardiovascular diseases (34), or other non-cancerous illnesses (35) may also alter miRNA profile and level, which should be taken into account by researchers when setting up control groups.

The past decade has seen remarkable achievements in the understanding of miRNAs in MM, including their various targets, effects, and dysregulation modes in disease development and progression. Some miRNAs, such as miR-20a, miR-19a, miR-92a, and miR-214-3p act as oncomiR playing important roles in anti-apoptosis, proliferation, migration, and invasion. Other miRNAs, including miR-15a-5p, miR203, miR144, miR199, miR-483-5p, miR-34a, miR-33b, miR-202, and miR-29a act as tumor suppressors. Also, some miRNAs are involved in the development of bone marrow microenvironment in MM; for example, miR143 and miR-29a promote angiogenesis and osteoblast differentiation (36, 37), miR199 neutralizes the oncogenic effect of bone marrow stromal cells (38), and miR-92a is essential for B cell development (39). These results provide valuable resources for the investigation of the etiology and treatment methods for MM. Interestingly, the role of miR-125b-5p in MM is still controversial since it has the ability to inhibit the growth and survival of MM cell and promote apoptosis and autophagy-associated cell death by targeting IRF4 and its downstream effector BLIMP-1 (40), but miR-125b-5p may generate counterproductive effects through different target genes or signaling pathways; it promotes MM progress by increasing p53 mRNA and protein and attenuating MM cell death in response to dexamethasone (41). Further exploration of miRNAs may enrich our perspective. More details are listed in **Table 6**.

**Table 6:** Summary of studies on the targets and functions of miRNAs included.

miRNA	Location	Targets	Functions of miRNA in multiple myeloma
miR-15a-5p	13q14.2	BCL-2, VEGF-A, PHF19, cyclin D1, cyclin D2, CDC25A	Cell growth suppression and apoptosis promotion (42, 43)
miR203	14q32.33	CREB1, VCAN	Inhibit myeloma cell proliferation (44).
miR143	5q32	HDAC7	Promotes angiogenesis and osteoblast differentiation (36).
miR144	17q11.2	c-MET, MEF2A, VCAN	Inhibits proliferation, angiogenesis, colony formation; promotes apoptosis (45, 46).
miR199	9q34.11	VCAN	Neutralizes the oncogenic effect of bone marrow stromal cells (38).
miR-125b-5p	11q24.1 or 21q21.1	IRF4, BLIMP-1, TP53	Inhibits the growth and survival of MM cell, promotes apoptosis and autophagy-associated cell death by targeting IRF4 and its downstream effector BLIMP-1 (40)
			Promotes MM progress by increasing p53 mRNA and protein, and attenuates MM cell death in response to dexamethasone (41).
miR-483-5p	11p15.5	ZNF197, ABCF2	Reduces cell viability, migration and colony formation (47).
miR-20a	13q31.3	PTEN, EGR2, SENP1, SOMO, cyclin D1	Promotes cell proliferation and migration. inhibits cell apoptosis and alters cell cycle (48–50).
miR-34a	1p36.22	c-MYC, BCL-2, NoTCH1, CDK6, TP53, SIRT1,	Promotes apoptosis and represses proliferation (51, 52).
miR-19a	13q31.3	SOCS-1, BIM, RHOB, CLTC, PSAP, PPP6R2	Inhibits apoptosis, promotes proliferation and migration (53, 54).
miR-92a	13q31.3	BIM	It’s essential for the development and survival of B cells, possess anti-apoptotic effect (39).
miR-214-3p	1q24.3	MCL1, PSMD10, ASF1B	Promotes MM progression by overexpression in myeloma fibroblasts (55).
miR-33b	17p11.2	PIM-1	Inhibits cell viability, migration, and colony formation (56).
miR-202	10q26.3	BAFF	Inhibits myeloma cell survival, growth, and adhesion (57).
miR-29a	7q32.3	VASH1, c-MYC	Promotes angiogenesis and osteogenesis (37), mediates anti-tumor activities in MM cells by targeting c-Myc (58).

MM, multiple myeloma.

Due to the important role of miRNAs in the tumorigenesis and progression of MM, circulating miRNAs have also shown their prognostic ability in MM, expanding their clinical application for identifying high-risk MM patients. As shown in **Table 7**, several studies have demonstrated that patients with different levels of miR-483-5p, miR-744, let-7e, miR-19a, miR-92a, miR-33b, miR-214, or miR-20a appeared with quite different survival rates.

**Table 7:** The prognostic value of circulating miRNAs in multiple myeloma.

Author	Sample size			miRNA	Expression with Poor prognosis	PFS	OS	Other outcomes
	Total	Low	High		
Qu X (18)	40	15	25	miR-483-5p	High	High: 15 months Low: 20 months p=0.025		Associated with ISS staging, p<0.05
	33	14	19	miR-20a	N/A	High: 16 months Low: 15 months p>0.05		
Kubiczkova L (19)	103	43	60	miR-744	Low		HR: 0.67 (95%CI, 0.55-0.82), p<0.0001	TTP: HR 0.69 (95%CI, 0.58-0.82), p<0.0001
	103	52	51	let-7e	Low		HR: 0.61 (95%CI, 0.42-8.83), p=0.002	TTP: HR 0.55 (95%CI, 0.42-0.72), p<0.0001
Hao M. (22)	103	45	58	miR-19a	Low	HR: 2.79 (95%CI, 1.42-5.47), p=0.003	HR: 2.99 (95%CI, 1.17-7.69), p=0.023	
Yoshizawa S (59)	90	62	28	miR-92a	Low	Low: 15.8 months High: 48 months p= 0.011		
Hao M (60)	158			miR-33b	High	Favorite PFS significantly	Favorite OS significantly	
	158			miR-19a	High	Favorite PFS significantly	Favorite OS significantly	
	158			miR-4254	N/A	Non-significance	Non-significance	Lower in healthy and complete remission patients compared to newly diagnosed and relapsed patients significantly
Hao M (61)	108			miR-135b	N/A	Non-significance	Non-significance	Correlated with grades of lytic bone lesions (r=0.404, p<0.001)
	108	35	73	miR-214	High	High: 8 months Low: 22 months p=0.015	High: 15 months Low: 28 months p=0.002	Correlated with grades of lytic bone lesions (r=0.455, p<0.0001).
Huang J (62)	28			miR-20a	High			RFS: P=0.01
Ren Y (63)	60	45	15	miR-720 + miR-1246	High	p<0.05		
Rocci A (64)	234			miR-720	N/A	HR: 0.89 (95%CI, 0.79-1.01), p=0.077	HR: 0.91 (95%CI, 0.77-1.07), p=0.26	

CI, confidence interval; EFS, event-free survival; HR, hazard ratio; MM, multiple myeloma; N/A, Not applicable; OS, overall survival; PFS, progression-free survival; RFS, Relapse-free survival; TTP, time to progression.

More interestingly, the expression levels of miR-19a, miR-744-5p, miR-143-5p, and miR-92a, had significantly different outcomes in bortezomib-based treatment (22, 65–67). Patients with higher serum levels of miR-214 had extended overall survival upon bisphosphonate-based therapy (68). In patients with autologous stem-cell transplantation, the lower level of miR-15a on day 0 was associated with the shorter time to engraftment, and miR-20a decreased at complete remission (69, 70). These results demonstrate that the expression patterns of circulating microRNAs are valuable markers for predicting treatment response. See **Table 8** for details.

**Table 8:** Application of circulating miRNAs in specific treatment of multiple myeloma.

Author	miRNA	Sample size	Treatment	Outcome
Jiang Y (17)	miR-125b-5p	Total: 35	Bortezomib, thalidomide, and dexamethasone, followed by thalidomide Maintenance	EFS: High, 8 months; low, 13 months, p=0.02
	miR-490-3p	Total: 35		EFS: High, 12 months; low, 13 months, p=0.23
Hao M. (22)	miR-19a	High level: 23 Low level: 30	Bortezomib-based	Patients with low miR-19a had significantly extended PFS (NR *vs*. 10.0 months), p=0.002
	miR-19a	High level: 28 Low level: 22	Thalidomide-based	Non-significance
Ren Y (63)	miR-720 + miR-1246	Decreased:28 Increased: 8	Bortezomib plus low-dose dexamethasone	Elevated levels were associated with worse PFS (p=0.0277)
		Decreased:16 Increased: 8	Vincristine, adriamycin, and dexamethasone	Elevated levels were associated with worse PFS (p=0.0184)
Robak P (65)	miR-744-5p	Refractory group: 19 Sensitive group:11	Bortezomib-based	Distribution difference, p=0.0006; predict refractoriness: OR=0.06, p=0.0146
	miR-143-5p			Distribution difference, p=0.0051; predict refractoriness: OR=4.14, p=0.0157
Narita D (66)	mir-92a	Newly diagnosed: 10; relapsed and/or refractory: 52	Bortezomib plus low-dose dexamethasone	Had higher expression in relapsed and/or refractory MM than in newly diagnosed MM, and correlated with chemotherapy response and disease progression
Yoshizawa S (67)	miR-92a	Total: 138	Bortezomib	Only up-regulated after therapy in responders
Hao M (68)	miR-214	Total: 108	Bisphosphonates	Higher level corelated with extended OS (NR *vs* 26.0 months, p=0.029)
Nowicki M (69)	miR-15a	Total: 42	Autologous hematopoietic stem cell transplantation	Patients with low expression on day 0 had a shorter time to engraftment than those with high expression (11 *vs* 13 days), p=0.01
Navarro A (70)	miR-20a	Total: 33	Autologous stem cell transplant	Expression at diagnosis was lower than complete remission, p= 0.009
Jung SH (71)	miR-19a	Good/poor responders: 19/19	Lenalidomide plus low-dose dexamethasone	Expressed between good responders and poor responders, p=0.073
	miR-20a			Between good responders and poor responders, p=0.241
Jasielec JK (72)	miR-199	Total: 30	Carfilzomib, lenalidomide, and dexamethasone	PFS: with decreased risk for progression, HR=0.41; p=0.04
Manier S (73)	let-7e	Total: 156	Bortezomib plus low-dose dexamethasone, followed by high-dose melphalan and autologous stem cell transplant	Low level, PFS: HR 2.01 (95%CI, 1.30-3.11), p=0.002; OS: HR 2.39 (95%CI, 1.09-5.24), p=0.030
	miR-125b			Low level, PFS: HR 1.02 (95%CI, 0.70-1.49), p=0.906; OS: HR 1.27 (95%CI, 0.60-2.72), p=0.533
	miR-15a			Low level, PFS: HR 1.37 (95%CI, 0.94-2.00), p=0.101; OS: HR 2.27 (95%CI, 1.02-5.06), p=0.046
	miR-19a			Low level, PFS: HR 0.13 (95%CI, 0.02-0.99), p=0.049
	miR-20a			Low level, PFS: HR 2.31 (95%CI, 1.52-3.53), p<0.001; OS: HR 2.91 (95%CI, 1.29-6.54), p=0.010
	miR-744			Low level, PFS: HR 1.32 (95%CI, 0.91-1.93), p=0.144; OS: HR 2.10 (95%CI, 0.97-4.53), p=0.059
	miR-92a			Low level, PFS: HR 1.39 (95%CI, 0.95-2.02), p=0.089; OS: HR 2.15 (95%CI, 1.00-4.65), p=0.051

CI, confidence interval; HR, hazard ratio; MM, multiple myeloma; NR: not reached; OR, odds ratio; OS, overall survival; PFS, progression-free survival.

### Strength and Limitations

The main strengths of this meta-analysis are the follows: 1) this is the first time that “cohort study design”, patient “stage or Ig isotype” and “newly diagnosed or untreated” information of MM patients were included in the subgroup analysis, summarizing the possible influencing factors of the current results; 2) it uses extensive but rigorous search strategies to optimize the quality of included literature; 3) it is stricter in assessing the quality of the included literature, providing a more detailed summary of included patients and healthy donors; 4) it structures a comprehensive review of the current understanding of circulating miRNAs in multiple myeloma, including the value of diagnosis, differential diagnosis, prognosis, and therapy-guiding. The major limitations are the following: 1) due to the insufficiency of miRNA studies included, the pooled diagnostic power of miRNA may be of some deviation, and the results of the subgroup analysis may also be biased to some extent; 2) some miRNAs are also dysregulated in other hematologic diseases; for example, miR-143, miR-144, and miR-199 are also under-expressed in child acute lymphoblastic leukemia, suggesting that circulating miRNAs may not be independent diagnostic biomarkers of MM, but can only be used as auxiliary and discriminatory diagnostic biomarkers (74).

### Recommendations

For diagnostic markers, randomized controlled trials are hard to achieve due to the limitations on patient choice and the invasiveness of the gold standard test; many studies have adopted case–control study designs. However, the reproducibility of miRNA-based studies in the diagnosis of MM is conducive to the feasibility of clinical application. Optimizing the flow of miRNA diagnostic tests according to the following suggestions would enable researchers to conduct systematic reviews and/or meta-analyses and draw more practical conclusions.

#### Patient Selection

“newly diagnosed or untreated patient” should be regarded as one of the criteria for the inclusion of diagnostic test, for circulating miRNA profile and level may change due to treatment. Pathological stage or IG isotype information of MM patients should be provided in detail, since the progression and Ig isotype of MM may affect circulating miRNA expression.

#### Study Design

Case–control study should not be perceived as a valid design for investigating the diagnostic value of miRNAs in MM patients. A cohort study may be a more applicable design initially, owing to the limited source of patients. Randomized controlled trials could be taken into consideration after the diagnostic power has been qualified.

#### Control Groups

To attenuate selection bias and avoid overestimation of diagnostic value, researchers should consider setting up control groups for suspected cases, individuals with MM precursor state, and patients with other conditions (such as inflammation, cardiovascular disease, or other non-cancerous conditions) separately, rather than just healthy controls. It was reported that about 28.6% of MM patients were diagnosed at the age of 65–74 years, and about 3.5% were under 44 (75). Besides, the incidence of MM is more prevalent in black race than in white, and higher in males than in females (76). Hence, the composition of age, ethnicity and gender of the control group should be consistent with that of the experimental group.

#### Sample Size

A sufficient sample size should be rigorously calculated in advance and can be achieved through collaboration between specialized institutions.

#### Specimen and Storage

Based on the long-term storage stability, circulating miRNAs have reliable performance as biomarkers. Both serum and plasma samples can be used to detect circulating miRNAs since there is no significant difference between serum or plasma-based tests. The researchers found that preservation at −20°C barely influenced the total amount of miRNAs for at least 2–4 years, with only slight changes in the concentration of individual miRNAs; in addition, storage at −80°C is even better (77).

#### Reference Standard

There are two widely used diagnostic criteria for patients with multiple myeloma and MUGS, one from the National Comprehensive Cancer Network (NCCN) and the other from the International Myeloma Working Group (IMWG) (78, 79). To avoid ambiguity and bias, researchers should clearly define the reference criteria.

#### Experimental Flow and Result Interpretation

Strict concealment measures should be observed throughout the study, including grouping, detection, and interpretation of test results. In addition the instruments, reagents, operating procedures, and the cutoff value of the test should be determined before the validation process and be detailed in the paper to avoid subjective bias and to ensure reproducibility. Except for the data on sensitivity, specificity and AUC, the direct presentation of true positive (TP), false positive (FP), true negative (TN), and false negative (FN) data would be of great benefit for future systematic evaluation.

## Conclusion

Through the unremitting efforts of researchers, miRNAs have been confirmed to be implicated in many pathophysiological processes of MM; however, the exact regulation mechanism remains to be fully elucidated. Much attention has been given to the diagnostic value of circulating miRNAs in MM over the past decade. This meta-analysis reveals that miR-4254 has the best potential to be a biomarker for MM diagnosis, and miRNA cluster might be a good choice to optimize the utilization. Successfully unraveling the diagnostic value of circulating miRNAs in MM will depend on multicenter large-scale studies with rigorous process design and a broad enrolment in patient and control groups.

## Data Availability Statement

The original data presented in the study were derived from the included article/supplementary material. Further inquiries can be directed to the corresponding author.

## Author Contributions

JZ designed the study, assessed the quality of the manuscript. YX and PX were responsible for study searching, data extraction and meta-analysis implementation. LZ reviewed the results of each step and resolved any differences in the evaluation opinions of other authors. YX, PX, and LZ co-drafted the manuscript. All authors contributed to the article and approved the submitted version.

## Funding

This project was supported by Youth Foundation of National Natural Science Foundation of China (81802075/H2003).

## Conflict of Interest

The authors declare that the research was conducted in the absence of any commercial or financial relationships that could be construed as a potential conflict of interest.
